# Supplementary material for: Retained introns in long RNA-seq reads are not reliably detected in sample-matched short reads
Source: Genome Biol. 2022 Nov 11;23:240. doi: 10.1186/s13059-022-02789-6 (PMC9652823; doi:10.1186/s13059-022-02789-6)
Supplement: Supplementary file 1 — Additional file 1. This file includes all supplementary figures and tables referenced in the article. [file 13059_2022_2789_MOESM1_ESM.pdf]

Additional File 1 of "Retained introns in long RNA-seq reads are not reliably detected in sample-matched short reads"

Julianne K. David[1,2,3†], Sean K. Maden[1,2,4†], Mary A. Wood[1,5,6], Reid F. Thompson[1,2,7,8,9*] and Abhinav Nellore[1,2,10*]

[1]Computational Biology Program, OHSU, Portland, OR USA.
[2]Dept. of Biomedical Engineering, OHSU, Portland, OR USA.
[3]Presently at Base5 Genomics, Inc., Palo Alto, CA USA.
[4]Presently at Dept. of Biostatistics, JHSPH, Baltimore, MD USA.
[5]Portland VA Research Foundation, Portland, OR USA.
[6]Presently at Phase Genomics, Inc., Seattle, WA USA.
[7]Dept. of Radiation Medicine, OHSU, Portland, OR USA.
[8]Dept. of Medical Informatics & Clinical Epidemiology, OHSU, Portland, OR USA.
[9]VA Portland Healthcare System, Portland, OR USA.
[10]Dept. of Surgery, OHSU, Portland, OR USA.

*Co-corresponding authors; thompsre@ohsu.edu; anellore@gmail.com;
†These authors contributed equally to this work.

Supplementary figures

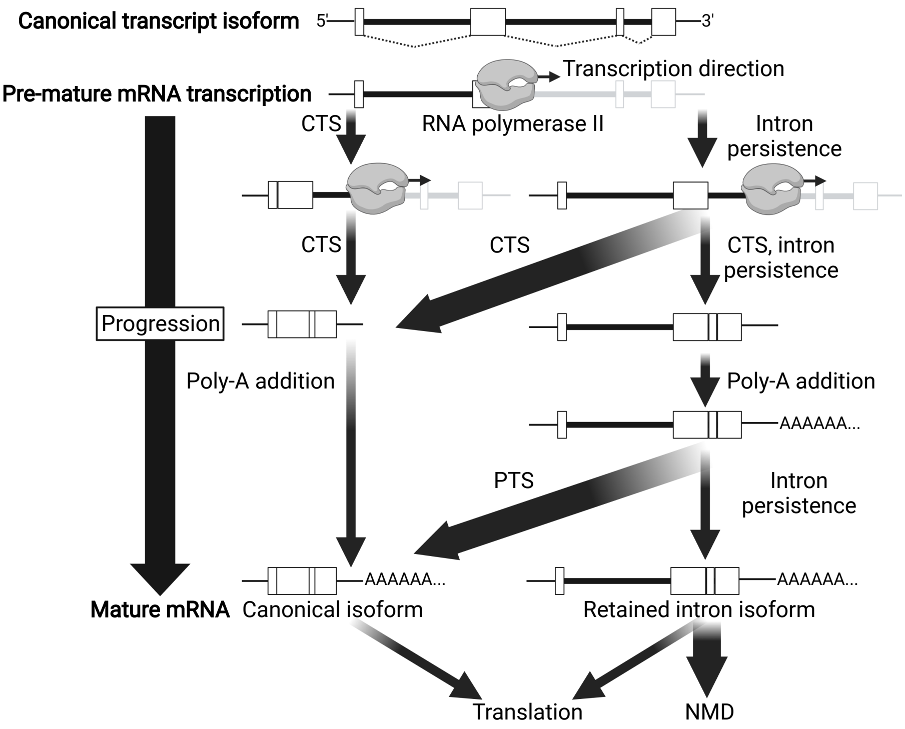

Fig. S1: Progression of transcript diagram, created with BioRender.com. Diagram depicts successive steps in transcript processing which progress from top to bottom. At the top is shown the presumed canonical transcript isoform with its expected splice pattern, followed by pre-mRNA processing steps, which branch between transcription by RNA polymerase II, co-transcriptional splicing (CTS), intron persistence, and poly(A) addition. At the bottom are the possible mature mRNA endpoints, including results from post-transcriptional mRNA splicing (PTS) and processing, which include translation and nonsense-mediated decay (NMD). Arrows are labeled with the events they represent, where arrow width sizes indicate their expected event frequencies.

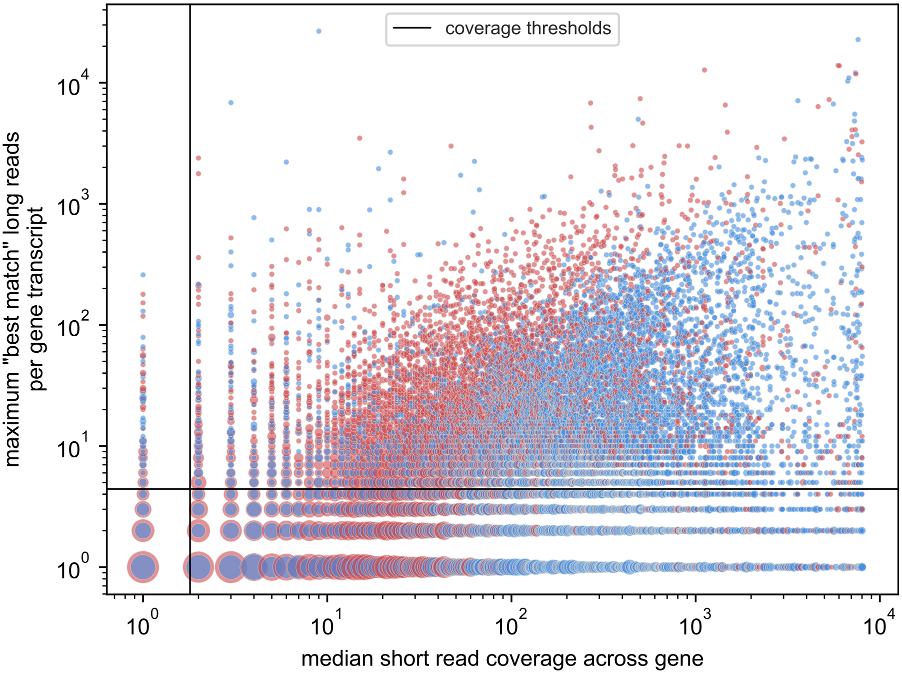

Fig. S2: Short- and long-read coverage of genes by sample. The maximum number of long reads assigned to one transcript of each gene (y-axis) vs. the median short-read coverage per base across the entire gene (x-axis) for HX1 (red) and iPSC (blue) samples, in log scale. The vertical line represents the minimum median short-read coverage (2) and the horizontal line represents the baseline minimum total long-read coverage per transcript (5) required for a gene to be included in our analysis; initial shortlist of genes considered are in the upper right region of the plot. Further filters on matched read quality were applied to generate the final subset of genes studied (see Methods section 4.6, Selection of target gene subset).

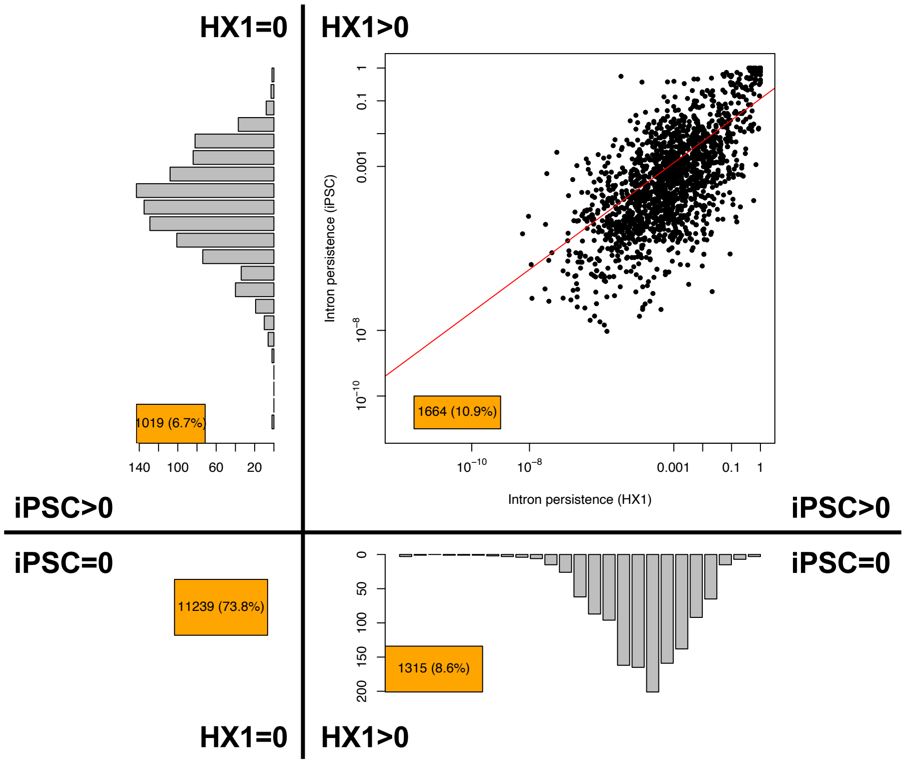

Fig. S3: Distribution of intron persistence values for introns in HX1 and iPSC samples. For introns included in both sample studies, bottom left quadrant represents introns with no persistence across both samples (73.8%), upper left represents introns with persistence in iPSC but not HX1 (6.7%), bottom right represents introns with persistence in HX1 but not iPSC (8.6%), and upper right is a scatterplot of persistences in iPSC (y-axis) vs. HX1 (x-axis) for introns with persistence in both (10.9%).

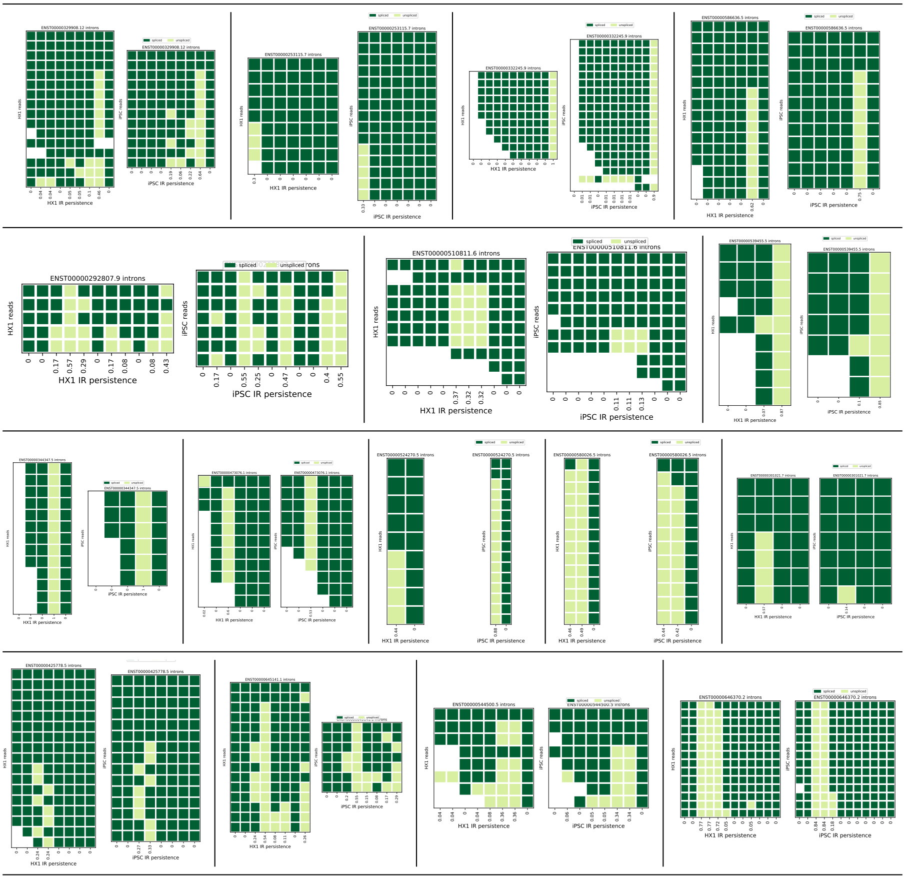

Fig. S4: Splicing similarity between samples. Heatmaps of splicing patterns for a selection of matched transcripts between HX1 and iPSC. Each subplot shows data for one transcript in HX1 (left) and iPSC (right) with rows representing transcript-matched long reads and columns representing transcript introns in 5' → 3' order. Transcripts were selected from a subset with 5–20 matched long reads in each sample and 5–20 introns. Dark green indicates a spliced out intron in a given read, light green indicates a retained intron, and white indicates no coverage of the intron in the read.

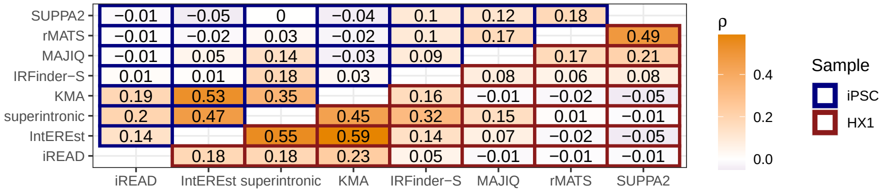

(a)

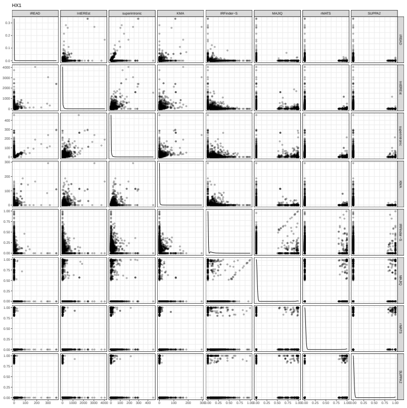

(b)

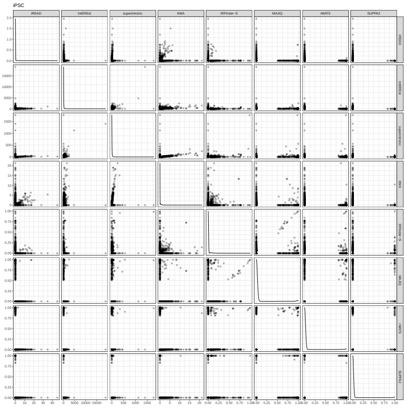

(c)

Fig. S5: (a) Pairwise correlations among the intron expression values output by eight short-read tools. Each element in this heatmap depicts the correlation in intron expression values (Spearman's test) between the indicated pair of short-read tools, as labeled along the x- and y-axes. Cell text indicates Spearman ρ coefficient, with corresponding color value obtained by the color gradient scale shown (from white to orange). Cell outline color indicates the sample for which inter-tool correlation was assessed (iPSC [top left] and HX1 [bottom right] are outlined in blue and red, respectively). (b) Intron expression scatter plots between all short-read IR-detection tool pairs (lower and upper triangles of plot grid) and density plots for each of the eight individual tools (diagonal plot grid) for HX1. (c) Intron expression scatter plots between all short-read IR-detection tool pairs (lower and upper triangles of plot grid) and density plots for each of the eight individual tools (diagonal plot grid) for iPSC.

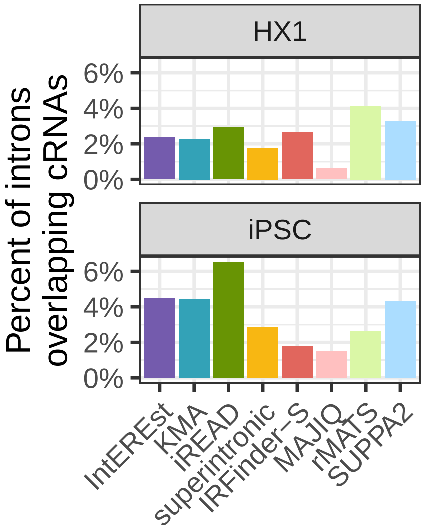

Fig. S6: cRNA overlap among called RIs for samples HX1 (top) and iPSC (bottom). Barplots quantify the percent of introns overlapping cRNAs (y-axes) across RIs called from eight short-read tools (x-axes).

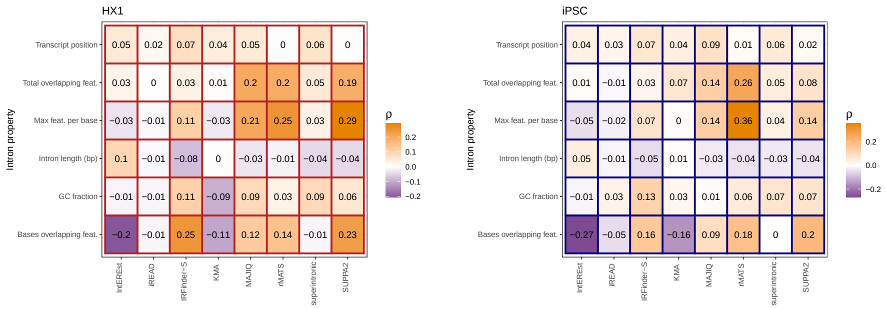

Fig. S7: Correlation of intron expression and continuous properties. Heatmap color fills and text show the Spearman ρ (purple = negative, white = near zero, orange = positive) between intron expression at eight short-read tools (x-axes/columns) and six continuous intron properties (y-axes/rows), for samples HX1 (left heatmap) and iPSC (right heatmap).

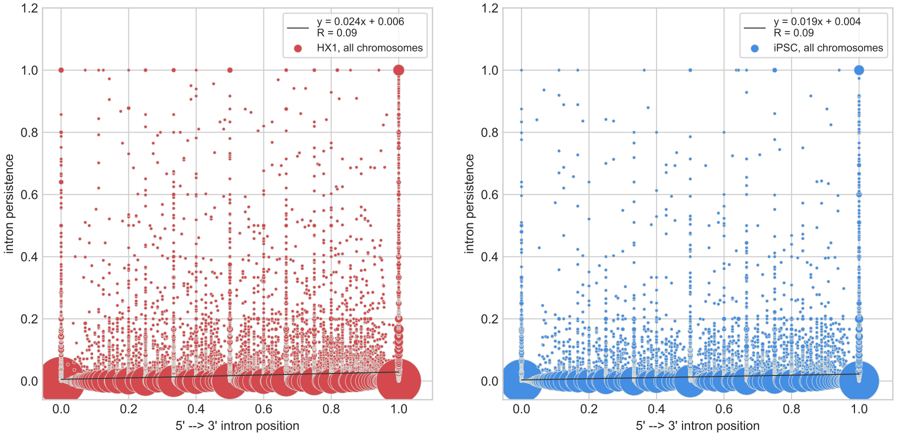

Fig. S8: Association of persistence with transcript position. Scatterplots of intron persistence vs. position within a transcript for HX1 (left, red), iPSC (right, blue). Each point represents one or more introns, with point size representing the number of points at each coordinate. Intron position is an intron-count normalized fraction where 0 represents the transcript's 5' end and 1 represents the 3' end. Plotted lines show the linear fit with equations show in the inset legends.

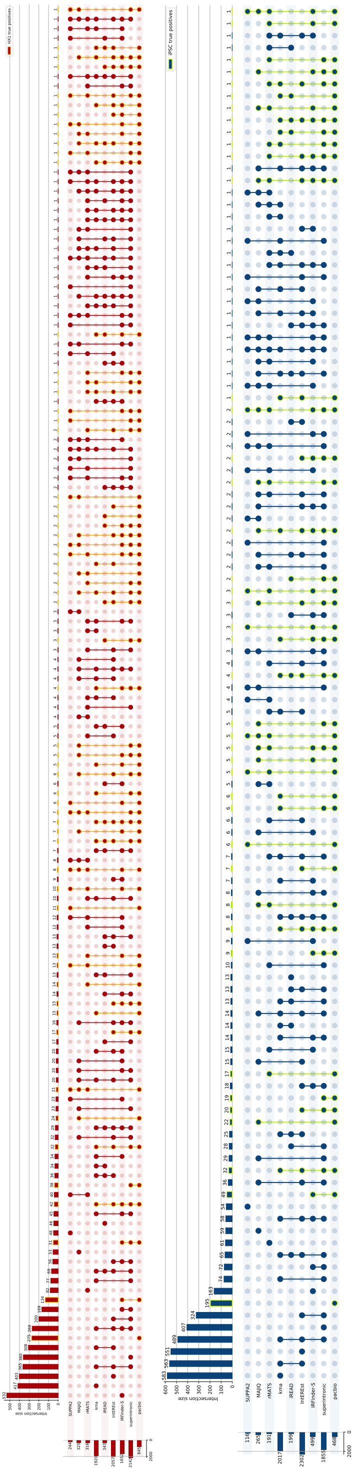

Fig. S9: Set overlaps of persistent introns and called RIs. Upset plots showing overlaps of sets of short-read called RIs and long read persistent introns for iPSC (above, blue) and HX1 (below, red). Sets of true positive persistent introns are highlighted in green for iPSC (above) and orange for HX1 (below).

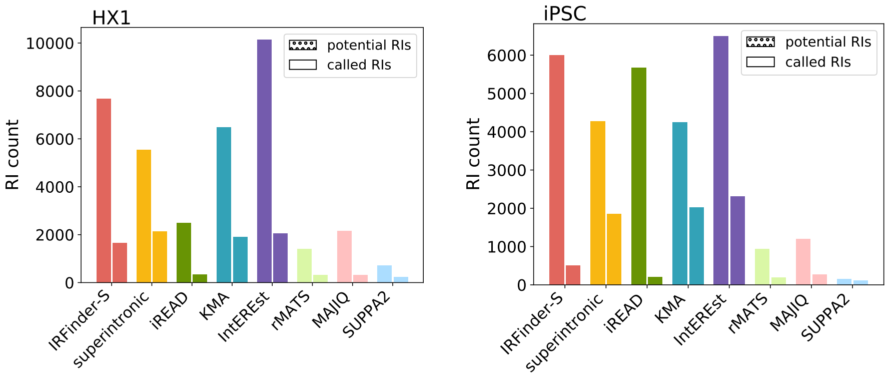

Fig. S10: Potential vs. called RI sets. For HX1 (left) and iPSC (right), counts of all potential (calculated nonzero expression) RIs (dotted hatch, left for each tool) and called (filtered) RIs (no hatch, right for each tool) for each SR detection tool (red = IRFinder-S, yellow = superintronic, green = iREAD, purple = IntEREst, blue = KMA, light green = rMATS, light pink = MAJIQ, and light blue = SUPPA2).

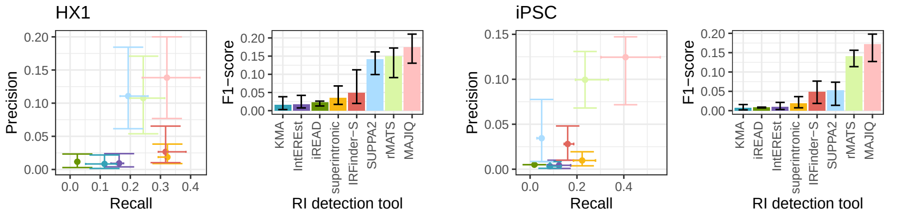

Fig. S11: Performance summaries across persistence cutoffs. Scatter plot y-axes show precision and x-axes show recall, and barplot y-axes show F1-scores, for samples HX1 (left plots) and iPSC (right plots). Colors indicate short-read RI detection tools (red = IRFinder-S, yellow = superintronic, green = iREAD, purple = IntEREst, blue = KMA, MAJIQ = light pink, rMATS = light green, SUPPA2 = light blue). Centroids and whiskers indicate the measure medians and interquartile ranges across persistence cutoffs varied from 0.1 to 0.9 at 0.1 intervals (Methods).

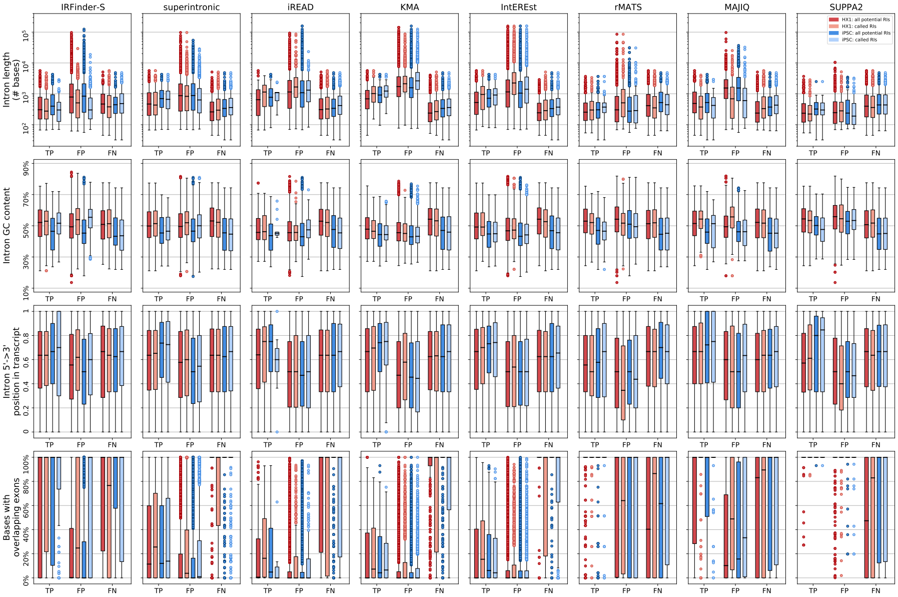

Fig. S12: Three target intron properties, length (top), % of bases in the intron that are G or C (2nd row), position along the direction of transcription (0 = 5', 1 = 3') (3rd row), and % of bases with an overlapping annotated exon (bottom) vs. TP, FP, and FN calls for HX1 and iPSC via potential RIs (darker) and called RIs (lighter), at long read persistence of 0.1 for 8 short read tools.

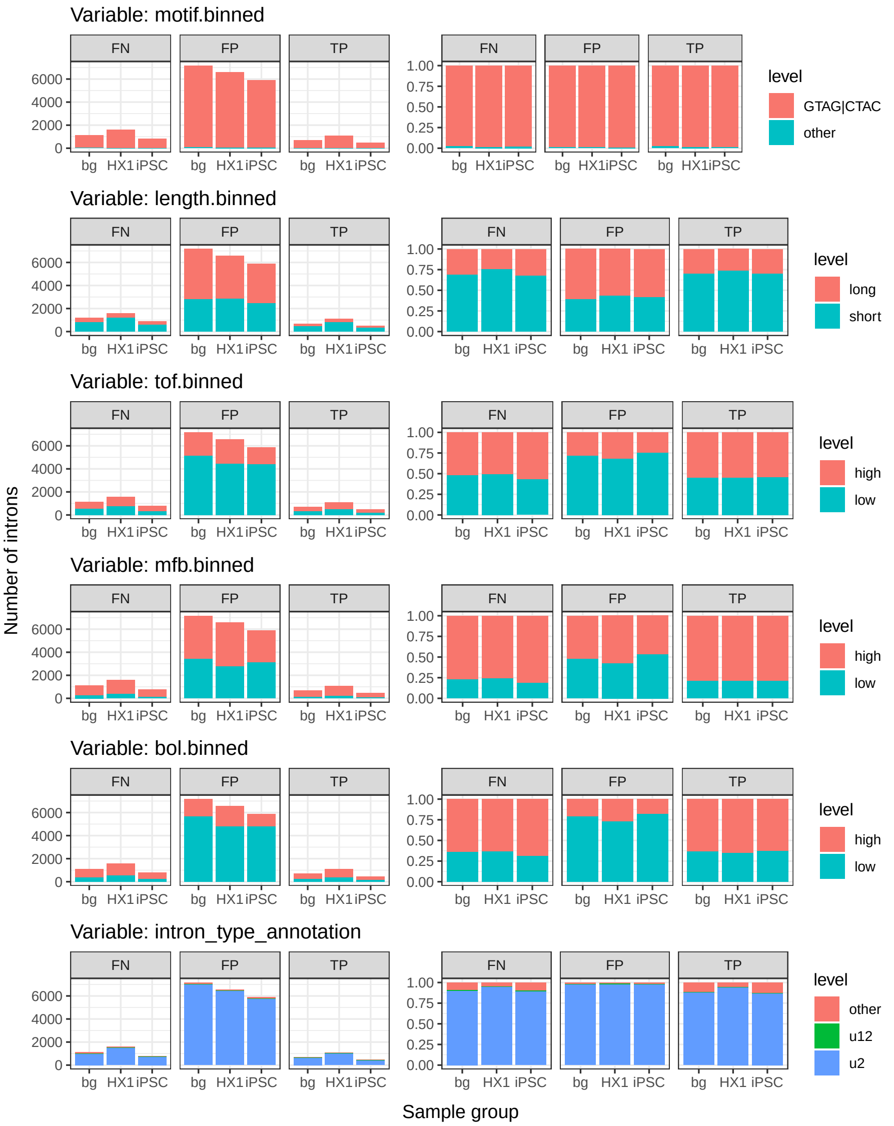

Fig. S13: Distributions of binned intron properties. Barplots of intron counts (left column) and percentages (right column) across unique levels (fill colors indicated in legends) for binned intron properties (plot titles). Results were binned by sample group types (columns, either HX1, iPSC, or the background of all unique introns) and intron 4+ truth metric categories TP, FP, and FN (ribbon labels, e.g. intron was TP in at least 4 tools for iPSC, etc. see Methods for definition details). Qualitative properties were binned by the top three most frequent levels (e.g. "intron type annotation" and "motif binned"), and quantitative properties were binned using the 50th quantile cutoff (e.g. "length", total overlapping features or "tof," max features per base or "mfb," and bases overlapped or "bol").

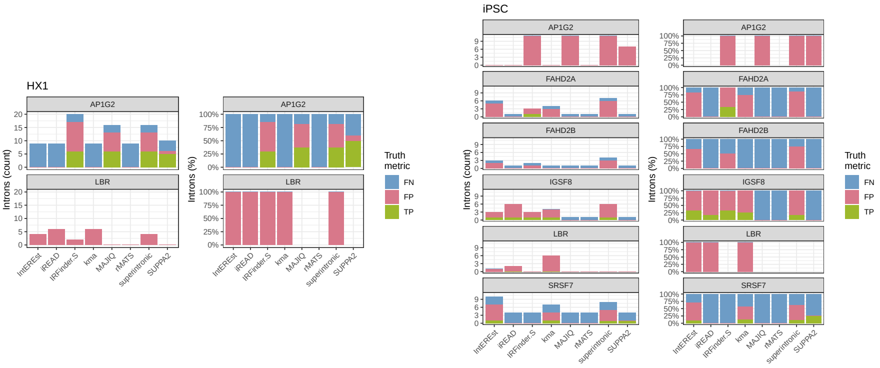

Fig. S14: Intron abundance by truth category across genes with validated RIs. Barplots show intron counts and percentages (y-axes) grouped by short-read tool (x-axes), gene (titles), for samples HX1 (left two plot columns) and iPSC (right two plot columns). Bar color fills indicate the short-read tool-specific truth category (green = TP, pink = FP, blue = FN, see Methods for category definition details).

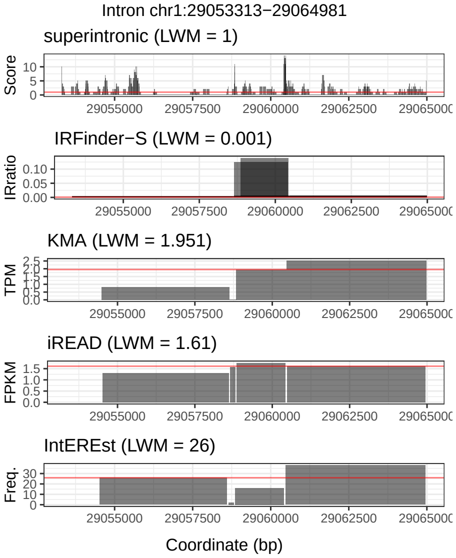

Fig. S15: Example length-weighted median expression (LWM) at intron *chr1*:29053313-29064981 (Methods). Intron expression (y-axes) is shown for genomic coordinates (x-axes), where expressed regions are represented by semi-transparent black rectangles which overlap the target intron. Results are grouped by each of five RI-specific short-read tools studied, and the LWM value calculated for each tool is shown in the plot titles and horizontal red lines.

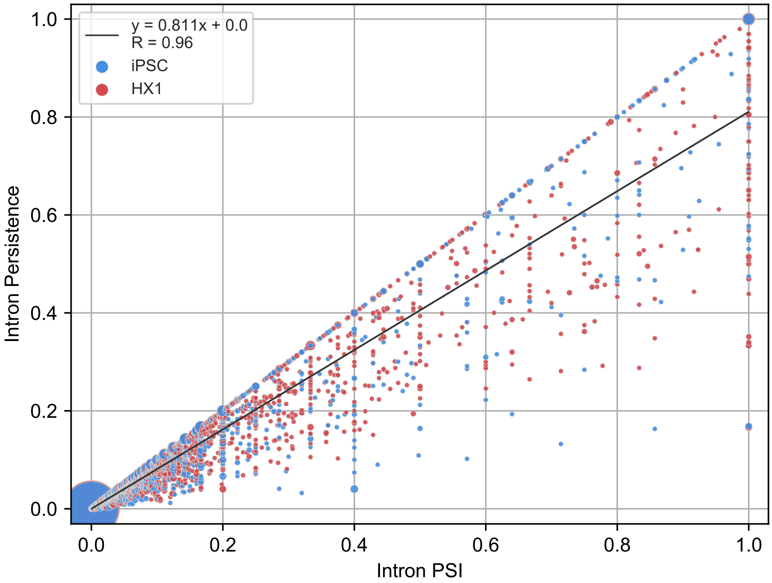

Fig. S16: Persistence (i.e., $\max_{t \in T_i} P_{i,t}$) vs. maximum intron PSI across transcripts (i.e., $\max_{t \in T_i}(R_{r,i}/|M^t|)$) in iPSC (blue) and HX1 (red).

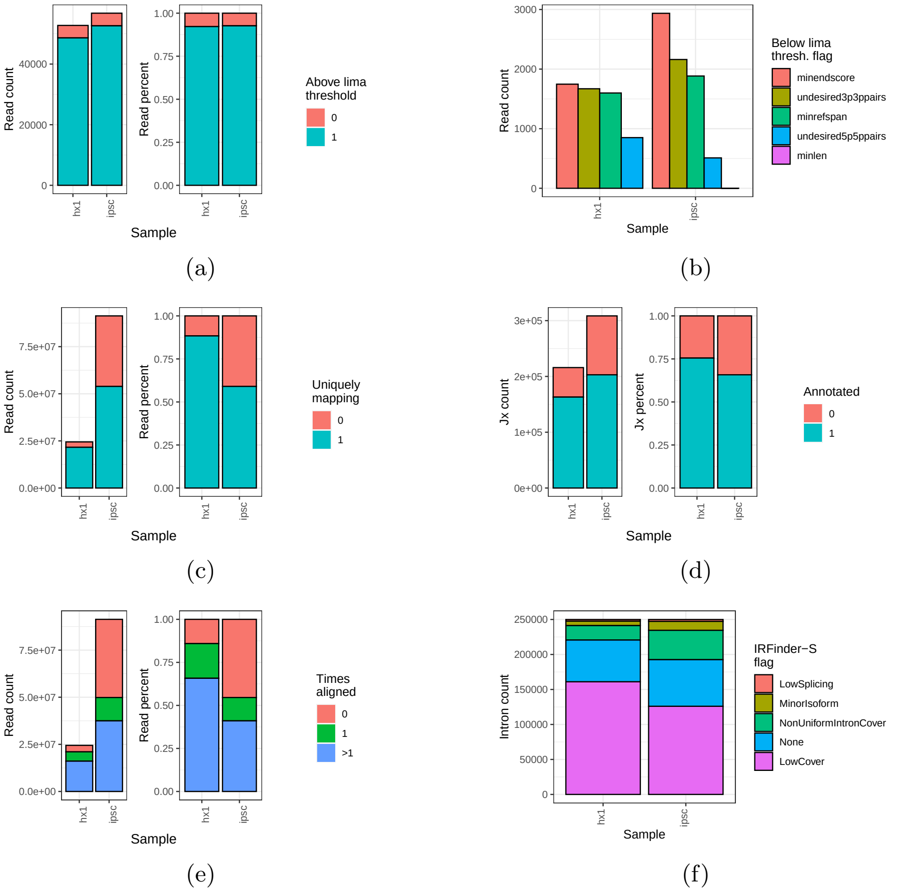

Fig. S17: Processing and alignment quality control. Results (y-axes, fill colors) across long-read (A-B) and short-read (C-F) data runs for the samples HX1 and iPSC (x-axes) as follows: (a) LIMA quality among long-reads. Barplot y-axes quantify long-read counts (left) and percentages (right) relative to the quality threshold (blue = above, pink = below), where medians across all runs are shown for iPSC. (b) LIMA flags among long-reads. Barplot y-axes quantify long-reads, where bar colors and x-axes indicate one of the five quality flags (magenta = below minimum length or "minlen", blue = undesired 5-prime 5-prime pairs as "undesired5p5ppairs", green = below reference span as "minrefspan", yellow = undesired 3-prime 3-prime pairs as "undesired3p3ppairs", and pink = below minimum end score as "minendscore"). (c) Unique mapping among STAR-aligned short-reads. Barplot y-axes quantify short-read counts (left) and percentages (right) by mappability (blue/1 = uniquely mapping, pink/0 = not uniquely mapping). (d) Annotation among STAR-aligned short-reads. Barplot y-axes as in (c) with color indicating annotation (blue/1 = annotated, pink/0 = not annotated). (e) Alignment counts among bowtie2-aligned short-reads. Barplot y-axes as in (c), where bar colors show alignment counts (blue = > 1 times, green = 1 time, pink = 0 times). (f) IRFinder-S flag quantities. Barplot y-axes as in (c), where bar colors show flag (magenta = low coverage as "LowCover", blue = none, green = non-uniform intron coverage as "NonUniformIntronCover", yellow = minor isoform presence as "MinorIsoform", pink = low splicing as "LowSplicing").

Supplementary tables

Sample name in paper	iPSC	HX1
Sample type	Induced pluripotent stem cell line	Whole blood (non-cancer)
Biosample ID	SAMN07611993	SAMN04251426
SRA Study ID	SRP098984	SRP065930
Long read platform	PacBio Iso-Seq RSII	PacBio Iso-Seq RSII
Size fractionated	No	Yes
Iso-Seq runs	27	46
Aligned long reads	839,558	945,180
Short-read platform	Illumina NextSeq 500	Illumina HiSeq 2000
Short read runs	1	1
Aligned short reads (% uniquely aligned)	91,330,785 (59%)	24,463,210 (88%)

Table S1: Description of sequencing data used in this paper.

sample	short read detection tool (RIs detected)		long read persistence threshold: >0	0.1	0.2	0.3	0.4	0.5	0.6	0.7	0.8	0.9
HX1		long read RI count	3219	845	416	274	186	140	86	58	41	25
	iREAD (343)	precision	0.370	0.082	0.029	0.023	0.017	0.012	0.006	0.003	0	0
		recall	0.039	0.033	0.024	0.029	0.032	0.029	0.023	0.017	0	0
		f-score	0.071	0.047	0.026	0.026	0.023	0.017	0.009	0.005	0	0
	IntEREst (2,057)	precision	0.314	0.071	0.039	0.024	0.016	0.009	0.007	0.004	0.002	0.001
		recall	0.200	0.173	0.195	0.182	0.177	0.136	0.163	0.138	0.098	0.080
		f-score	0.245	0.101	0.066	0.043	0.029	0.017	0.013	0.008	0.004	0.002
	superintronic (2,142)	precision	0.495	0.127	0.063	0.040	0.028	0.019	0.013	0.009	0.006	0.003
		recall	0.329	0.323	0.327	0.310	0.328	0.286	0.326	0.328	0.293	0.240
		f-score	0.395	0.183	0.106	0.070	0.052	0.035	0.025	0.017	0.011	0.006
	kma (1,920)	precision	0.305	0.068	0.035	0.022	0.013	0.008	0.005	0.002	0.001	0.001
		recall	0.182	0.154	0.163	0.157	0.134	0.114	0.105	0.052	0.049	0.040
		f-score	0.228	0.094	0.058	0.039	0.024	0.016	0.009	0.003	0.002	0.001
	IRFinder-S (1,652)	precision	0.585	0.197	0.100	0.065	0.042	0.027	0.014	0.010	0.008	0.003
		recall	0.300	0.385	0.397	0.394	0.371	0.314	0.267	0.293	0.317	0.200
		f-score	0.397	0.260	0.160	0.112	0.075	0.049	0.026	0.020	0.015	0.006
	rMATS (316)	precision	0.554	0.316	0.237	0.171	0.127	0.108	0.085	0.054	0.041	0.032
		recall	0.054	0.118	0.180	0.197	0.215	0.243	0.314	0.293	0.317	0.400
		f-score	0.099	0.172	0.205	0.183	0.159	0.149	0.134	0.091	0.073	0.059
	MAJIQ (325)	precision	0.569	0.302	0.240	0.200	0.175	0.138	0.111	0.077	0.062	0.043
		recall	0.057	0.116	0.188	0.237	0.306	0.321	0.419	0.431	0.488	0.560
		f-score	0.104	0.168	0.211	0.217	0.223	0.194	0.175	0.131	0.109	0.080
	SUPPA2 (244)	precision	0.660	0.361	0.254	0.184	0.139	0.111	0.090	0.061	0.045	0.037
		recall	0.050	0.104	0.149	0.164	0.183	0.193	0.256	0.259	0.268	0.360
		f-score	0.093	0.162	0.188	0.174	0.158	0.141	0.133	0.099	0.077	0.067
iPSC		long read RI count	2379	468	208	130	106	81	55	35	27	19
	iREAD (199)	precision	0.302	0.045	0.015	0.005	0.005	0.005	0.005	0.005	0.005	0.005
		recall	0.025	0.019	0.014	0.008	0.009	0.012	0.018	0.029	0.037	0.053
		f-score	0.047	0.027	0.015	0.006	0.007	0.007	0.008	0.009	0.009	0.009
	IntEREst (2,302)	precision	0.237	0.036	0.016	0.010	0.006	0.004	0.002	0.001	0.001	0
		recall	0.230	0.177	0.173	0.177	0.132	0.123	0.073	0.057	0.074	0
		f-score	0.233	0.060	0.029	0.019	0.012	0.008	0.003	0.002	0.002	0
	superintronic (1,855)	precision	0.414	0.071	0.032	0.019	0.013	0.010	0.005	0.004	0.003	0.002
		recall	0.323	0.282	0.284	0.277	0.236	0.222	0.182	0.200	0.185	0.158
		f-score	0.363	0.114	0.057	0.036	0.025	0.019	0.010	0.007	0.005	0.003
	kma (2,017)	precision	0.190	0.033	0.014	0.008	0.004	0.003	0.002	0.001	0.001	0.000
		recall	0.161	0.143	0.135	0.131	0.085	0.086	0.073	0.057	0.074	0.053
		f-score	0.175	0.054	0.025	0.016	0.008	0.007	0.004	0.002	0.002	0.001
	IRFinder-S (499)	precision	0.623	0.196	0.084	0.048	0.034	0.028	0.016	0.010	0.006	0.004
		recall	0.131	0.209	0.202	0.185	0.160	0.173	0.145	0.143	0.111	0.105
		f-score	0.216	0.203	0.119	0.076	0.056	0.048	0.029	0.019	0.011	0.008
	rMATS (191)	precision	0.644	0.288	0.173	0.136	0.120	0.099	0.073	0.068	0.047	0.042
		recall	0.052	0.118	0.159	0.200	0.217	0.235	0.255	0.371	0.333	0.421
		f-score	0.096	0.167	0.165	0.162	0.155	0.140	0.114	0.115	0.083	0.076
	MAJIQ (265)	precision	0.502	0.238	0.177	0.151	0.140	0.125	0.091	0.072	0.057	0.045
		recall	0.056	0.135	0.226	0.308	0.349	0.407	0.436	0.543	0.556	0.632
		f-score	0.101	0.172	0.199	0.203	0.199	0.191	0.150	0.127	0.103	0.085
	SUPPA2 (116)	precision	0.655	0.216	0.103	0.078	0.060	0.034	0.009	0.009	0.009	0
		recall	0.032	0.053	0.058	0.069	0.066	0.049	0.018	0.029	0.037	0
		f-score	0.061	0.086	0.074	0.073	0.063	0.041	0.012	0.013	0.014	0

Table S2: Performance metrics for called RIs across persistence thresholds. Green indicates the highest value per threshold and sample for each metric.

Gene	Source	Intron coordinates	Discovery assay	Validation assay	Disease or cell type association	Samples with gene expression	Sample intron persistence
AP1G2	Jeong 2021 [1]	chr14:23565702-23565815 (intron 5)	short-read RNA-seq	RT-PCR	mesenchymal stem cell	HX1, iPSC	0.06, 0
FAHD2A	Li 2021 [2]	chr2:95412765-95412894	short-read RNA-seq	Nanostring	Alzheimer's disease	iPSC	0.06
FAHD2B	Li 2021 [2]	chr2:97083818-97083947	short-read RNA-seq	Nanostring	Alzheimer's disease	iPSC	0.05
IGSF8	Li 2021 [2]	chr1:160094172-160094868	short-read RNA-seq	Nanostring	Alzheimer's disease	iPSC	0.02
LBR	Wong 2013 [3]	chr1:225410417-225411336 (intron 9)	short-read RNA-seq	RT-PCR, RNA-seq	granulocyte	HX1, iPSC	0.02, 0.01
SRSF7	Lejeune 2001 [4]	chr2:38748654-38749528 (intron 3)	*in vitro* splicing assays	Northern blot	–	iPSC	0.17

Table S3: Properties and sources of experimentally validated RIs studied.

References

[1] Jeong, Ji-Eun and Seol, Binna and Kim, Han-Seop and Kim, Jae-Yun and Cho, Yee-Sook. Exploration of alternative splicing events in mesenchymal stem cells from human induced pluripotent stem cells. *Genes* **12** (5), 737 (2021) .

[2] Li, Hong-Dong and Funk, Cory C and McFarland, Karen and Dammer, Eric B and Allen, Mariet and Carrasquillo, Minerva M and Levites, Yona and Chakrabarty, Paramita and Burgess, Jeremy D and Wang, Xue and others. Integrative functional genomic analysis of intron retention in human and mouse brain with Alzheimer's disease. *Alzheimer's & Dementia* **17** (6), 984–1004 (2021) .

[3] Wong, Justin J-L and Ritchie, William and Ebner, Olivia A and Selbach, Matthias and Wong, Jason WH and Huang, Yizhou and Gao, Dadi and Pinello, Natalia and Gonzalez, Maria and Baidya, Kinsha and others. Orchestrated intron retention regulates normal granulocyte differentiation. *Cell* **154** (3), 583–595 (2013) .

[4] Lejeune, Fabrice and Cavaloc, Yvon and Stevenin, James. Alternative splicing of intron 3 of the serine/arginine-rich protein 9G8 gene: Identification of flanking exonic splicing enhancers and involvement of 9G8 as a trans-acting factor. *Journal of Biological Chemistry* **276** (11), 7850–7858 (2001) .
